# Supplementary material for: Identification and Characterization of Variants in Intron 6 of the LPL Gene Locus among a Sample of the Kuwaiti Population
Source: Genes (Basel). 2022 Apr 9;13(4):664. doi: 10.3390/genes13040664 (PMC9024856; doi:10.3390/genes13040664)
Supplement: Supplementary file 1 [file genes-13-00664-s001.zip › genes-1620950-supplementary.pdf]

Supplementary Material

Figure S1: Nucleotide sequence of amplified regions of intron 6 of the *LPL* gene.

(A) Region 1

<u>Primer 62 binding sequence: 498bp</u>

Forward primer

ACTAAACCCTGAGCCCTGGTGTTTCTGTTGATAGGGGGTTGCATTGATCCATTTGTCTGAGGCTTCTAAT
TCCCATTGTCAGCAAGGTCCCAGTGCTCAGTGTGGGATTTGCAGCCTTGCTCGCTGCCCTCCCCTGTAAA
TGTGGCCATTAGCATGGGCTAGGCTATCAGCACAGAGCTCAGAGCTCATTTGGAACCATCCACCTCGGGT
CAACAAACTATAACCCTTGTGCCAAATCCAGCCTACTTCCTGCTTTTGTAAATAGTTTTTTTAAAACTTT
TAAGTTCAGGGGTACGTATGTAGGTTTGCTAAAAAGGTAAACTTGTGACATGGGAGTTTGTTGTCCAGAA
TATTCCATCACCCAGGTATTAAGCTTAGTACCCATTAGTTACTTTTCCTGAAGCTCTCCCTCCTCCCACC
CTCTGGGAGGCCCCAGTGTCTGTTGTTCCCCTCTATGTGCTCATGCAAAGTTTTATTAGGACACAGCCAC
ACACATTC

Reverse primer

(B) Region 2

<u>Primer 69 binding sequence: 571bp</u>

Forward primer

TGGCCTCCAAGAACTCTTTTTTCCTCCATCATCATGGTTCTATTTTAGTCCTGCTGCCTTTCCTTTTAAC
CTCTCCCCAGGCCCATTTGCTCAGGGTTTTTGGTAGAGACCAGAGGAGGGGCAGGGAGGAGATATAGAAG
TTCAACTACCTGCTTCCAGAGGCTGTCCCTAGTATAGAATACTTTAGGGGCTGGCTTTACAAGGCAGTCC
TTGTGGCCTCACTGATGGCTCAATGAAATAAGTTCTTTTTTAAAAAAAATTTTATTTATTTCCATAGGTT
ATTGGGGGAACAGGTGGTGTTTGGTTACATGAGTAAGTTCTTTAGTAGTGATTTGTGAGATTTTGGTGTG
CCCATTACGGAATGGAAAAATCAACGAAATAAGTTCTATGATGCACCTACTAGACACCTAATCTGCGCTA
GATGGTGGGGGAATTAAGAGCATGGGCATGATCCTGTGACCGGAAGCCCGCTTACAGTCAGGGTGGAGGA
CAGACCTACTCATGAAACAAACACAGTGACATATAGTGACACAGAAGCAAATGTCAAATATGCTTGCTCC
AGATGCTAAGG

Reverse primer

Table S1. The quantities of reagents used in making the PCR master mix for the amplification of two regions of intron 6 of the *LPL* gene.

**Reagents and components**	**Volume (µl)**
Double Distilled water (ddH_2O)	2.5
Applied Biosystems AmpliTaq Gold® *Master Mix*	10
Forward Primer (0.26um)	1.25
Reverse Primer (0.26um)	1.25
Diluted DNA (10ng/µl)	5
Total	25

Table S2. Thermal cycler parameters for the amplification of two regions of intron 6 of the *LPL* gene.

**Parameter**	**Temperature (°C)**	**Time**	**Number of cycles**
Initial denaturation	94	5 minutes	1
Denaturation	94	30 seconds	35
Annealing	65	30 seconds	
Extension	72	1 minute	
Final extension	72	7 minutes	1
Hold	4	∞	-

Table S3. The quantities of reagents used in making the PCR master mix for the sequencing of two regions of intron 6 of the *LPL* gene.

**Reagents and components**	**Volume (μl)**
Double Distilled water (ddH_2O)	1.5
BigDye® Terminator v1.1/3.1 Sequencing Buffer (5X)	2
BigDye® Terminator v3.1 Ready Reaction Mix	2
Forward Primer or reverse primer (0.26um)	2
Diluted DNA (10ng/μl)	2.5
Total	10

Table S4. The thermal cycler parameters for the sequencing of two regions of intron 6 of the *LPL* gene.

**Parameter**	**Temperature (°C)**	**Time**	**Number of cycles**
Initial denaturation	96	1 minutes	1
Denaturation	96	10 seconds	25
Annealing	50	5 seconds	
Extension	60	1:15 minute	
Hold	4	∞	-
